# Leveraging artificial intelligence for clinical decision support in personalized standard regimen recommendation for cancer

**DOI:** 10.1186/s40779-025-00617-z

**Published:** 2025-06-19

**Authors:** Ya-Le Jiang, Guo Zhao, Shu-Hang Wang, Ning Li

**Affiliations:** Clinical Trial Center, National Cancer Center/National Clinical Research Center for Cancer/Cancer Hospital, Chinese Academy of Medical Sciences and Peking Union Medical College, Beijing, 100021 China

**Keywords:** Artificial intelligence (AI), Precision medicine, Standard of care

Dear Editor,

The incorporation of artificial intelligence (AI) into clinical treatment is becoming a revolutionary influence, altering the ways in which various disease types, such as cancer, are diagnosed, treated, and managed [1]. We summarized the status of AI-driven precision oncology in Additional file 1, and highlighted the transformative potential of AI in cancer therapy in Additional file 1: Table S1. AI-driven precision oncology studies have primarily focused on targeted therapy, as this area lends itself well to the strengths of AI in clear actionability and inherent personalization based on multiple-omic data [2]. While progress is significant, challenges like data standardization and equitable access to targeted therapies remain to be resolved. When the application of AI extends beyond targeted therapy, data complexity is even more challenging. Treatments such as immunotherapy and chemotherapy require integrating diverse data types, including imaging, clinical, and immune profiles. For numerous non-targeted therapies, predictive biomarkers are less well-defined, making it harder but more urgent for AI to identify certain patterns. Combination therapy and clinical trial matching are emerging focuses, with AI identifying synergistic drug combinations and potentially fitted trials.

## Dilemma of AI application: data learnability and model usage

The application of AI in cancer treatment is mainly restricted to two aspects. Firstly, the learnability of current training data is not powerful enough, which is usually confined to clinical knowledge and cross-sectional omics data of patients at a certain time point. As a result, the models may fail to fully capture the dynamic changes in tumor characteristics during the treatment process. Thus, AI can hardly overcome the impact of factors such as tumor heterogeneity on treatment regimen selection, and it cannot surpass human understanding of tumors. Secondly, these powerful models have not yet explored the most suitable modes in their application to medical fields, so AI may not truly exert its advantages in reasoning and learning.

Generative AI emerged as a disruptive technology two years ago. These techniques, such as large language models (LLMs), are much more powerful than many expected. It was estimated that more than 80% of physicians in America are using AI in their work, but whether its utilization could improve clinical efficacy is still debatable [3]. The reasoning abilities of LLMs were compared against physicians, showing better performance in processing medical data and clinical reasoning [4]. Conversely, in a randomized clinical trial, the availability of an LLM did not significantly enhance diagnostic reasoning performance among 50 physicians, compared with using conventional resources [5].

In addition to the specific advantages and disadvantages mentioned above, the general limitations of current AI models in medical practice should be noted. First, decision-making is restricted in certain therapy types, which cannot cover the majority of circumstances faced by oncologists. They may need to seek different models for different patients. Second, longitudinal clinical and multiple-omic data for individual patients are lacking in these trials, which could not provide sufficient evidence for AI models to explore the patterns of patients’ responses to therapeutic effects. As a result, it is also impossible to understand sequential decisions on therapies given the prior treatment history in cancer patients. Third, the intrinsic feelings and values of patients have not been incorporated into the intricate decision-making model algorithm. There remains a pressing need to develop a more patient-centric, intellectualized treatment paradigm.

With the revolutionary rapid iteration of technology, Chatbots themselves are also improving dramatically. It does beg the question: what are the right roles for humans and computers, and how should we bring the two together to optimize their power? The AI should not be solely used as a search engine, but to obey its nature, acting as adjuncts in clinical decision-making, and offering insights that clinicians may overlook through its remarkable learning and reasoning ability. Oncologists must also rely on their clinical judgment, patient-centered communication, and multidisciplinary collaboration to make the best treatment decisions.

## Overlooked unmet needs in standard therapy selection

Standard therapies are those accepted by medical experts as a proper treatment for a certain type of disease and that is widely used by health care professionals. Clinical guidelines and expert consensus are the primary references for clinicians in regimen selection. Among these, guidelines are supported by a higher level of evidence and thus considered more authoritative than the latter. 

Selecting a standard therapy from clinical guidelines is a critical part of patient care, but it comes with several challenges for a clinician. The first stems from the complexity of cancer biology. The heterogeneity of tumors causes vastly different genetic, molecular, and microenvironmental characteristics, which may not be accounted for by guidelines. Although some subtypes, like human epidermal growth factor receptor 2 (HER2)-positive breast cancer, have been well-defined towards specific therapies, not all patients fit neatly into these categories. Even in those better-defined cancer types, incomplete biomarker testing due to cost, tissue availability, or lack of access to advanced diagnostics may impede clinician’s application of guidelines. Additionally, guidelines have inherent limitations. Their multi-year update cycles lag behind rapidly emerging therapies and evidence, risking outdated recommendations. They frequently list multiple treatment options without prioritization strategies. Conflicting recommendations across guidelines [e.g., National Comprehensive Cancer Network (NCCN), European Society for Medical Oncology (ESMO), American Society of Clinical Oncology (ASCO), Chinese Society of Clinical Oncology (CSCO)] for identical cancer stages require physicians to adapt based on local resources and expertise, leaving individualized optimization uncertain. Critically, population-based guidelines neglect individual factors. Comorbidities and performance status may exclude guideline-recommended therapies. Non-medical considerations—patient priorities (quality of life vs. aggressive treatment), and cost concerns (especially in resource-limited areas)—must be integrated into clinical decisions. The selection of standard treatments with AI assistance is one of the potential approaches to solving this complex problem, given its strengths in knowledge graph construction, multi-dimensional information processing, and data-based inference. Nevertheless, AI has not been applied appropriately and thoroughly in this field.

## A prosperous solution to precision medicine ecosystem: AI combined with real-world evidence

**Fig. 1:**
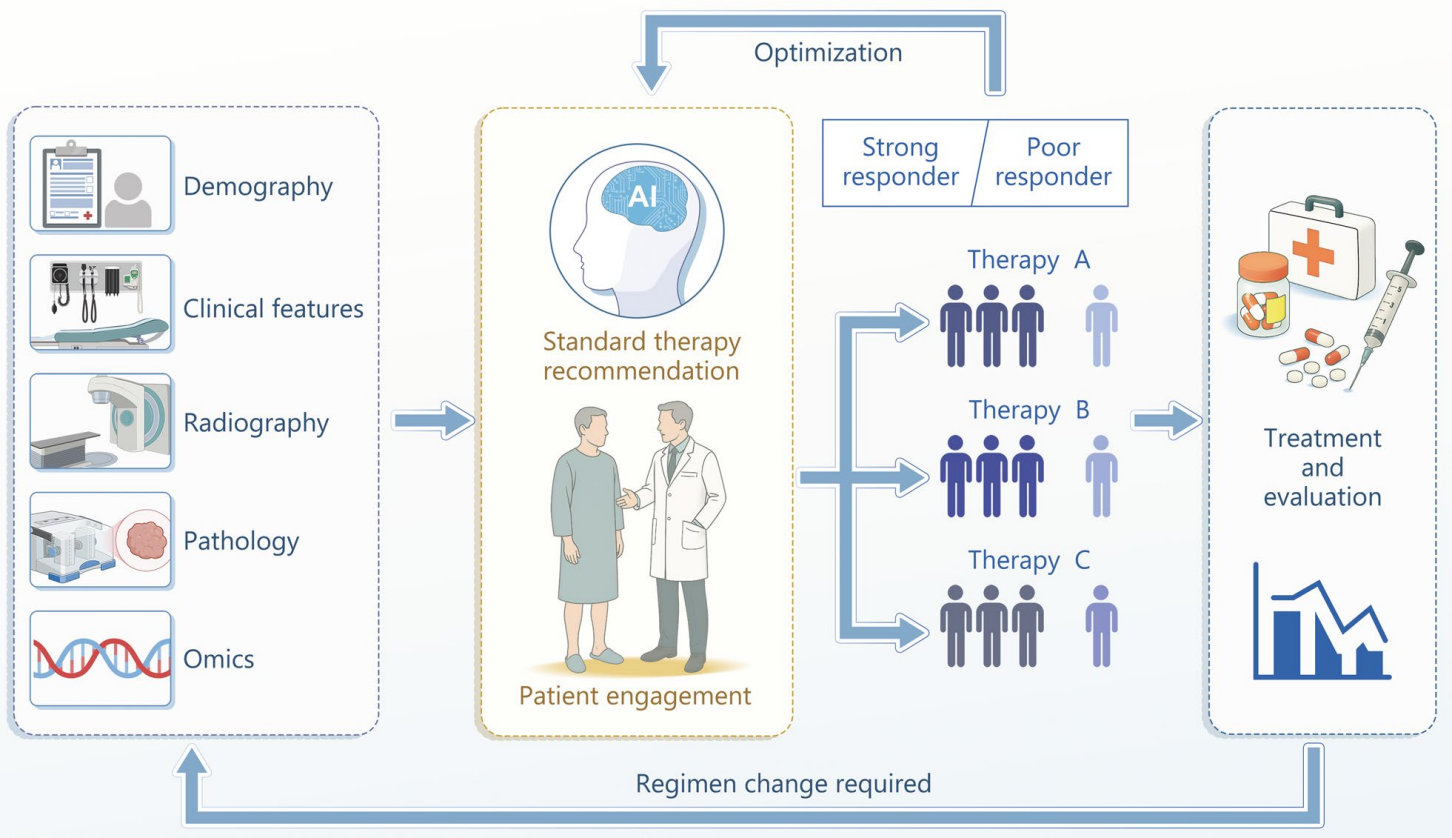
Scheme of the SINGULARITY study, an exploratory real-world study aiming to evaluate the feasibility of an AI-guided standard treatment selection model for advanced solid tumors. Multiple dimensional data including demographics, clinical information (such as pathology, tumor staging, imaging, previous treatment responses), and multi-omics data will be collected for a multi-agent system based on AI models to recommend standard treatment options. The final regimen will be jointly selected by the patient and clinician, thereby delivering a personalized treatment. AI artificial intelligence

Multimodality AI combined with real-world evidence is believed to serve as an appropriate solution to intellectual assistance in standard therapy selection [6]. Therefore, the National Cancer Center in China has initiated a real-world. Study to INvestigate optimal standard treatment selection for tumor patients GUided by bioLogicAlly-infoRmed multI-agenT sYstem (NCT06824792). The SINGULARITY study aims to utilize multimodal AI, incorporating genomic, imaging, clinical, real-world data, and patient preferences and values to refine treatment recommendations and improve standard therapy selection across all cancer treatment modalities (Fig. 1). Real-world data will be collected adhering to rigorous standards of clinical trials to form high-level evidence. The AI model will provide precise guidance on the selection of the next-step treatment regimen based on complete and real-time updated individual patient data, including omics (specific data not disclosed). Compared with other AI models for treatment recommendation, this study focuses on the full-cycle combination of multi-omic data and real-world data to make recommendations based on current guidelines. We intended to tackle the critical challenge of the inadequate guiding accuracy inherent in the existing guidelines and present meticulously tailored options of optimized standard treatment to patients. The as-yet-unfulfilled medical need for individualized oncological standard treatment is promising to be satisfied in the precision oncology ecosystem, enhancing the overall quality and effectiveness of cancer care.

## Supplementary Information

**Additional file 1.** Status of various artificial intelligence (AI) models for cancer treatment. **Table S1** Summary of AI-driven applications related to regimens optimization in cancer treatment.

## Data Availability

Not applicable.

## Abbreviations

AI: Artificial intelligence
ASCO: American Society of Clinical Oncology
CSCO: Chinese Society of Clinical Oncology
ESMO: European Society For Medical Oncology
HER2: Human epidermalgrowth factor receptor 2
LLM: Large language models
NCCN: National Comprehensive Cancer Network
