# Supplementary material for: Leveraging artificial intelligence for clinical decision support in personalized standard regimen recommendation for cancer
Source: Mil Med Res. 2025 Jun 19;12:31. doi: 10.1186/s40779-025-00617-z (PMC12177993; doi:10.1186/s40779-025-00617-z)
Supplement: Supplementary file 1 — Additional file 1. Status of various artificial intelligence (AI) models for cancer treatment. Table S1 Summary of AI-driven applications related to regimens optimization in cancer treatment. [file 40779_2025_617_MOESM1_ESM.pdf]

Status of various artificial intelligence (AI) models for cancer treatment

The integration of AI in oncology has advanced significantly across therapeutic domains, though challenges persist. IBM Watson for Oncology is an artificial intelligence assistant decision system based on National Comprehensive Cancer Network (NCCN) guidelines and empirical evaluation, which has achieved a high concordance rate with a multidisciplinary team approach in medical oncology, especially for breast cancer [1]. Foundation Medicine and Oncotype IQ leverage genomic profiling and multi-omics data to predict drug responses, yet their utility is constrained by incomplete biomarker testing and tumor microenvironment variability, as highlighted in studies exploring chromatin accessibility and transcriptional heterogeneity in vascular smooth muscle cell (VSMC) proliferation models [2]. The NCI-MATCH Trial employs AI to match rare mutations with targeted therapies, but its impact is limited by biomarker accessibility and the dynamic nature of genomic evidence, reflecting broader challenges in preclinical model design and validation [3].

For immunotherapy, MD Anderson's AI Platform utilizes natural language processing (NLP) to analyze tumor microenvironment data from pathology reports, demonstrating success in predicting checkpoint inhibitor responses in melanoma and non-small cell lung cancer (NSCLC). However, its scalability is hampered by validation gaps in low-resource settings and cost barriers [4]. Tempus dynamically integrates real-world data (RWD) to identify immunotherapy candidates, though discrepancies between guidelines [e.g., NCCN vs. European Society For Medical Oncology (ESMO)] complicate clinical implementation, mirroring issues observed in transcriptional network rewiring studies of injury-responsive VSMCs [5].

In chemotherapy optimization, AI models for ovarian cancer predict platinum sensitivity and optimize carboplatin-paclitaxel regimens, yet face challenges from tumor microenvironment variability and limited validation in recurrent cases [6]. Similarly, breast cancer chemotherapy models

balance efficacy and toxicity using reinforcement learning, but their clinical translation requires addressing patient-specific factors such as comorbidities and performance status, as seen in studies of VSMC clonal expansion and drug resistance mechanisms [7].

Radiotherapy planning tools like Varian Medical Systems and RaySearch Laboratories employ deep learning for automated contouring and adaptive dose optimization, reducing planning time [8, 9]. However, their effectiveness depends on high-quality imaging data and lags behind rapid advancements in tumor biology understanding, such as chromatin accessibility changes in proliferating VSMCs.

In combination therapy design, in silico Medicine uses generative AI for drug repurposing and synergistic target identification [e.g., programmed cell death ligand 1 (PD-L1)/Aurora kinase A (AURKA) inhibitors in triple-negative breast cancer], though clinical translation is hindered by biomarker complexity and regulatory network redundancies [10]. The American Association of Cancer Research (AACR) Project Genomics Evidence Neoplasia Information Exchange (GENIE) is an international data-sharing consortium based on real-world evidence for targeted therapy, by linking genomic data to clinical outcomes. The database is expected to serve as a powerful tool for precision cancer medicine research and treatment option selection [11]. Besides, Huang et al. [12] introduced TxGNN, a graph foundation model for zero-shot drug repurposing, identifying therapeutic candidates even for diseases with limited treatment options or no existing drugs.

For clinical trial matching, Trialjectory [13] and Antidote Match [14] utilize natural language processing (NLP) to align patient electronic health records (EHRs) with trial criteria, yet struggle with incomplete data and eligibility interpretation. Flatiron Health [15] accelerates trial recruitment through structured/unstructured EHR analysis but is constrained by regional healthcare infrastructure disparities, paralleling challenges in global biomarker validation and preclinical study design

standardization.

Overall, while AI models enhance precision oncology by bridging molecular insights and clinical practice, their optimal application requires addressing tumor heterogeneity, guideline discrepancies, and real-world validation gaps, as underscored by both preclinical model studies and clinical trial design research.

Table S1 Summary of AI-driven applications related to regimens optimization in cancer treatment

**Therapy type**	**Key focus**	**Examples of AI application studies**	**Reference**
Targeted therapy	Genomic analysis, biomarker identification, mutation-driven therapy recommendations	IBM Watson for Oncology	[1]
		Foundation Medicine and Oncotype IQ	[2]
		NCI-MATCH Trial	[3]
Immunotherapy	Predicting immunotherapy response, neoantigen prediction, immune profiling	MD Anderson's AI Platform	[4]
		Tempus	[5]
Chemotherapy	Optimizing regimens, predicting resistance, reducing toxicity	AI models for ovarian cancer	[6]
		Breast cancer chemotherapy optimization	[7]
Radiation therapy	Treatment planning, outcome prediction, dose optimization	Varian Medical Systems	[8]
		RaySearch Laboratories	[9]
Combination therapies	Identifying synergistic drug combinations, overcoming resistance	In Silico Medicine	[10]
		Project GENIE	[11]
		TxGNN	[12]
Clinical trial matching	Matching patients to trials based on genomic and clinical data	Trialjectory	[13]
		Antidote Match	[14]
		Flatiron Health	[15]

AI artificial intelligence, *GENIE* Genomics Evidence Neoplasia Information Exchange, *NCI-MATCH* The National Cancer Institute–Molecular Analysis for Therapy Choice

References

1. Somashekhar SP, Sepúlveda MJ, Puglielli S, Norden AD, Shortliffe EH, Rohit Kumar C, et al. Watson for Oncology and breast cancer treatment recommendations: agreement with an expert multidisciplinary tumor board. Ann Oncol. 2018;29(2):418-23.
2. Karol D, McKinnon M, Mukhtar L, Awan A, Lo B, Wheatley-Price P. The impact of foundation medicine testing on cancer patients: a single academic centre experience. Front Oncol. 2021;11:687730.
3. Flaherty KT, Gray RJ, Chen AP, Li S, McShane LM, Patton D, et al. Molecular landscape and actionable alterations in a genomically guided cancer clinical trial: National Cancer Institute Molecular Analysis for Therapy Choice (NCI-MATCH). J Clin Oncol. 2020;38(33):3883-94.
4. Business Wire: Generate: biomedicines and MD anderson enter co-development and commercialization agreement to accelerate novel protein therapeutics for oncology using generative AI. 2023. https://www.businesswire.com/news/home/20230425006147/en/GenerateBiomedicines-and-MD-Anderson-Enter-Co-Development-and-Commercialization-Agreement-to-Accelerate-Novel-Protein-Therapeutics-for-Oncology-Using-Generative-AI. Accessed 08 Apr 2025.
5. Tempus. https://www.tempus.com/. Accessed 08 Apr 2025.
6. Crispin-Ortuzar M, Woitek R, Reinius MAV, Moore E, Beer L, Bura V, et al. Integrated radiogenomics models predict response to neoadjuvant chemotherapy in high-grade serous ovarian cancer. Nat Commun. 2023;14(1):6756.
7. Sabir Z, Munawar M, Abdelkawy MA, Raja MAZ, Ünlü C, Jeelani MB, et al. Numerical investigations of the fractional-order mathematical model underlying immune-chemotherapeutic treatment for breast cancer using the neural networks. Fractal Fract. 2022;6(4):184.
8. Varian Medical System. https://www.varian.com/. Accessed 14 Apr 2025.
9. RaySearch Laboratories. https://www.raysearchlabs.cn/. Accessed 14 Apr 2025.
10. Jeon M, Kim S, Park S, Lee H, Kang J. In silico drug combination discovery for personalized cancer therapy. BMC Syst Biol. 2018;12(Suppl 2):16.

11. AACR Project GENIE Consortium. AACR project GENIE: powering precision medicine through an international consortium. Cancer Discov. 2017;7(8):818-31.

12. Huang K, Chandak P, Wang Q, Havaldar S, Vaid A, Leskovec J, et al. A foundation model for clinician-centered drug repurposing. Nat Med. 2024;30(12):3601-13.

13. Trialjectory. https://www.leal.health/. Accessed 14 Apr 2025.

14. Antidote Match. https://www.antidote.me/. Accessed 14 Apr 2025.

15. Flatiron Health. https://flatiron.com/. Accessed 08 Apr 2025.
